# Supplementary material for: Anti-Müllerian hormone and fertility in women after childhood cancer treatment: Association with current infertility risk classifications
Source: PLoS One. 2024 Aug 12;19(8):e0308827. doi: 10.1371/journal.pone.0308827 (PMC11318921; doi:10.1371/journal.pone.0308827)
Supplement: S1 Table — (DOCX) [file pone.0308827.s001.docx]

**S1 Table. Cancer diagnoses and treatments.**

**S1a Table. Childhood cancer survivors (CCSs) with different diagnoses and treatments divided into infertility risk groups according to Table 1a.**

| **Group** | **No risk** | | **Low-risk** | | **Moderate-risk** | | **High-risk** | | **Very high-risk** | **Only surgery** | **Unilateral oophorectomy** | **All CCSs** |
| --- | --- | --- | --- | --- | --- | --- | --- | --- | --- | --- | --- | --- |
| N | 17 | 31 | | 29 | | 37 | | 13 | | 19 | 13 | 167 (%) |
| **Diagnoses** |  | |  | |  | |  | |  |  |  |  |
| Leukaemia | 0 | | 19 | | 13 | | 8 | | 10 | 0 | 1 | 51 (31%) |
| Brain tumour | 15 | | 0 | | 0 | | 3 | | 0 | 18 | 0 | 39 (23%) |
| Lymphoma | 0 | | 1 | | 2 | | 12 | | 3 | 0 | 1 | 21 (13%) |
| Sarcoma | 1 | | 1 | | 10 | | 5 | | 0 | 0 | 1 | 18 (11%) |
| Wilms tumour | 0 | | 9 | | 0 | | 8 | | 0 | 0 | 0 | 19 (11%) |
| Ovarian tumour | 0 | | 0 | | 0 | | 0 | | 0 | 0 | 10 | 11 (7%) |
| Other | 1 | | 1 | | 4 | | 1 | | 0 | 1 | 0 | 8 (5%) |
| **Treatment** |  |  | |  | |  | |  | |  |  |  |
| Cytotoxic treatment | 2 | | 31 | | 29 | | 35 | | 12 | 0 | 13 | 127 (76%) |
| Alkylating agents | 0 | | 0 | | 29 | | 27 | | 10 | 0 | 13 | 81 (49%) |
| Radiotherapy, abdominal | 0 | | 0 | | 0 | | 15 | | 9 | 0 | 4 | 34 (20%) |
| Radiotherapy, cranial | 14 | | 6 | | 9 | | 13 | | 7 | 0 | 1 | 53 (32%) |
| TBI | 0 | | 0 | | 0 | | 0 | | 7 | 0 | 0 | 7 (4%) |
| HSCT | 0 | | 0 | | 0 | | 0 | | 11 | 0 | 0 | 11 (7%) |

From the cohort of 167 CCSs, eight were excluded from subgrouping, six of whom had inconclusive data and two underwent bilateral oophorectomy.
TBI: total body irradiation; HSCT: hematopoietic stem cell transplantation.

**S1b Table. Childhood cancer survivors (CCSs) with different diagnoses and treatments divided into infertility risk groups according to Table 1b.**

| **Group** | | **1** | | | | **2** | | **3** | | **4** | | **All CCSs** | |
| --- | --- | --- | --- | --- | --- | --- | --- | --- | --- | --- | --- | --- | --- |
| N | | | | 54 | | 13 | | 48 | | 50 | | 167 (%) | |
| **Diagnoses** | |  | | | |  | |  | |  | |  | |
| Leukaemia | | 13 | | | | 1 | | 23 | | 14 | | 51 (31%) | |
| Brain tumour | | 18 | | | | 0 | | 15 | | 6 | | 39 (23%) | |
| Lymphoma | | 1 | | | | 1 | | 8 | | 10 | | 21 (13%) | |
| Sarcoma | | 6 | | | | 1 | | 2 | | 9 | | 18 (11%) | |
| Wilms tumour | | 9 | | | | 0 | | 0 | | 10 | | 19 (11%) | |
| Ovarian tumour | | 0 | | | | 10 | | 0 | | 0 | | 11 (7%) | |
| Other | | 7 | | | | 0 | | 0 | | 1 | | 8 (5%) | |
| **Treatment** | | | |  | |  | |  | |  | |  | |
| Cytotoxic treatment | | 35 | | | | 13 | | 33 | | 44 | | 127 (76%) | |
| Alkylating agents | | 8 | | | | 13 | | 27 | | 32 | | 81 (49%) | |
| Radiotherapy, abdominal | | 0 | | | | 4 | | 1 | | 29 | | 34 (20%) | |
| Radiotherapy, cranial | | 0 | | | | 1 | | 32 | | 20 | | 53 (32%) | |
| TBI | | 0 | | | | 0 | | 0 | | 7 | | 7 (4%) | |
| HSCT | | 0 | | | | 0 | | 0 | | 11 | | 11 (7%) | |

From the cohort of 167 CCSs, two were excluded from subgrouping due to bilateral oophorectomy.
TBI: total body irradiation; HSCT: hematopoietic stem cell transplantation.
