# Supplementary material for: Anti-Müllerian hormone and fertility in women after childhood cancer treatment: Association with current infertility risk classifications
Source: PLoS One. 2024 Aug 12;19(8):e0308827. doi: 10.1371/journal.pone.0308827 (PMC11318921; doi:10.1371/journal.pone.0308827)
Supplement: S1 File — (DOCX) [file pone.0308827.s002.docx]

**S1 File. Linear regression analyses.**

**AMH and age at examination.**

| **Model Summary** | | | | |
| --- | --- | --- | --- | --- |
| Model | R | R Square | Adjusted R Square | Std. Error of the Estimate |
| 1 | ,463^a^ | ,215 | ,210 | 2,8713 |
| a. Predictors: (Constant), Subjects, Age at examination | | | | |

| **ANOVA^a^** | | | | | | |
| --- | --- | --- | --- | --- | --- | --- |
| Model | | Sum of Squares | df | Mean Square | F | Sig. |
| 1 | Regression | 734,803 | 2 | 367,402 | 44,563 | <,001^b^ |
|  | Residual | 2687,750 | 326 | 8,245 |  |  |
|  | Total | 3422,553 | 328 |  |  |  |
| a. Dependent Variable: AMH ng/ml | | | | | | |
| b. Predictors: (Constant), Subjects, Age at examination | | | | | | |

| **Coefficients^a^** | | | | | | | | |
| --- | --- | --- | --- | --- | --- | --- | --- | --- |
| Model | | Unstandardized Coefficients | | Standardized Coefficients | t | Sig. | 95,0% Confidence Interval for B | |
|  |  | B | Std. Error | Beta |  |  | Lower Bound | Upper Bound |
| 1 | (Constant) | 8,878 | ,654 |  | 13,586 | <,001 | 7,593 | 10,164 |
|  | Age at examination | -,165 | ,018 | -,462 | -9,408 | <,001 | -,199 | -,130 |
|  | Subjects | -,386 | ,317 | -,060 | -1,218 | ,224 | -1,010 | ,237 |
| a. Dependent Variable: AMH ng/ml | | | | | | | | |

**Swedish infertility risk groups <40 years.**

| **Model Summary** | | | | |
| --- | --- | --- | --- | --- |
| Model | R | R Square | Adjusted R Square | Std. Error of the Estimate |
| 1 | ,302^a^ | ,091 | ,063 | 3,2672 |
| a. Predictors: (Constant), Risk_group=unilateral oophorectomy, Risk_group=very high-risk, Risk_group=only surgery, Risk_group=no risk, Risk_group=moderate risk, Risk_group=low risk, Risk_group=high-risk | | | | |

| **ANOVA^a^** | | | | | | |
| --- | --- | --- | --- | --- | --- | --- |
| Model | | Sum of Squares | df | Mean Square | F | Sig. |
| 1 | Regression | 240,221 | 7 | 34,317 | 3,215 | ,003^b^ |
|  | Residual | 2391,181 | 224 | 10,675 |  |  |
|  | Total | 2631,402 | 231 |  |  |  |
| a. Dependent Variable: AMH ng/ml | | | | | | |
| b. Predictors: (Constant), Risk_group=unilateral oophorectomy, Risk_group=very high-risk, Risk_group=only surgery, Risk_group=no risk, Risk_group=moderate risk, Risk_group=low risk, Risk_group=high-risk | | | | | | |

| **Coefficients^a^** | | | | | | | | |
| --- | --- | --- | --- | --- | --- | --- | --- | --- |
| Model | | Unstandardized Coefficients | | Standardized Coefficients | t | Sig. | 95,0% Confidence Interval for B | |
|  |  | B | Std. Error | Beta |  |  | Lower Bound | Upper Bound |
| 1 | (Constant) | 4,014 | ,306 |  | 13,116 | <,001 | 3,411 | 4,617 |
|  | Risk_group=no risk | ,903 | ,992 | ,059 | ,911 | ,363 | -1,051 | 2,857 |
|  | Risk_group=low risk | ,402 | ,710 | ,038 | ,566 | ,572 | -,997 | 1,801 |
|  | Risk_group=moderate risk | ,609 | ,734 | ,055 | ,830 | ,408 | -,837 | 2,055 |
|  | Risk_group=high-risk | -1,962 | ,689 | -,190 | -2,847 | ,005 | -3,320 | -,604 |
|  | Risk_group=very high-risk | -3,449 | 1,078 | -,208 | -3,201 | ,002 | -5,573 | -1,326 |
|  | Risk_group=only surgery | ,174 | 1,032 | ,011 | ,169 | ,866 | -1,859 | 2,207 |
|  | Risk_group=unilateral oophorectomy | -,784 | 1,272 | -,040 | -,616 | ,538 | -3,291 | 1,723 |
| a. Dependent Variable: AMH ng/ml | | | | | | | | |

Adjusted for age at examination.

| **Model Summary** | | | | |
| --- | --- | --- | --- | --- |
| Model | R | R Square | Adjusted R Square | Std. Error of the Estimate |
| 1 | ,380^a^ | ,145 | ,114 | 3,1768 |
| a. Predictors: (Constant), Age at examination, Risk_group=very high-risk, Risk_group=only surgery, Risk_group=no risk, Risk_group=unilateral oophorectomy, Risk_group=low risk, Risk_group=high-risk, Risk_group=moderate risk | | | | |

| **ANOVA^a^** | | | | | | |
| --- | --- | --- | --- | --- | --- | --- |
| Model | | Sum of Squares | df | Mean Square | F | Sig. |
| 1 | Regression | 380,885 | 8 | 47,611 | 4,718 | <,001^b^ |
|  | Residual | 2250,518 | 223 | 10,092 |  |  |
|  | Total | 2631,402 | 231 |  |  |  |
| a. Dependent Variable: AMH ng/ml | | | | | | |
| b. Predictors: (Constant), Age at examination, Risk_group=very high-risk, Risk_group=only surgery, Risk_group=no risk, Risk_group=unilateral oophorectomy, Risk_group=low risk, Risk_group=high-risk, Risk_group=moderate risk | | | | | | |

| **Coefficients^a^** | | | | | | | | |
| --- | --- | --- | --- | --- | --- | --- | --- | --- |
| Model | | Unstandardized Coefficients | | Standardized Coefficients | t | Sig. | 95,0% Confidence Interval for B | |
|  |  | B | Std. Error | Beta |  |  | Lower Bound | Upper Bound |
| 1 | (Constant) | 7,848 | 1,069 |  | 7,340 | <,001 | 5,741 | 9,955 |
|  | Risk_group=no risk | ,762 | ,965 | ,050 | ,789 | ,431 | -1,140 | 2,663 |
|  | Risk_group=low risk | ,168 | ,693 | ,016 | ,242 | ,809 | -1,198 | 1,534 |
|  | Risk_group=moderate risk | ,224 | ,721 | ,020 | ,311 | ,756 | -1,197 | 1,645 |
|  | Risk_group=high-risk | -1,713 | ,673 | -,166 | -2,544 | ,012 | -3,040 | -,386 |
|  | Risk_group=very high-risk | -3,498 | 1,048 | -,211 | -3,339 | <,001 | -5,563 | -1,433 |
|  | Risk_group=only surgery | ,080 | 1,003 | ,005 | ,080 | ,936 | -1,897 | 2,057 |
|  | Risk_group=unilateral oophorectomy | -,298 | 1,244 | -,015 | -,239 | ,811 | -2,749 | 2,154 |
|  | Age at examination | -,126 | ,034 | -,238 | -3,733 | <,001 | -,193 | -,060 |
| a. Dependent Variable: AMH ng/ml | | | | | | | | |
