# Supplementary material for: Anti-Müllerian hormone and fertility in women after childhood cancer treatment: Association with current infertility risk classifications
Source: PLoS One. 2024 Aug 12;19(8):e0308827. doi: 10.1371/journal.pone.0308827 (PMC11318921; doi:10.1371/journal.pone.0308827)
Supplement: Attachment — Submitted filename: Response to Reviewers.docx [file pone.0308827.s003.docx]

PONE-D-24-15437

Anti-Müllerian hormone and fertility in women after childhood cancer treatment: association with current infertility risk classifications

Dear Academic Editor Antoine Naem and reviewers,

Thank you for your valuable comments on the manuscript and for the opportunity to submit a revised version. Our reply is below marked with red text, addressing the journal requirements as well as reviewers' comments.

Kind regards,

Anna Nyström & the co-authors

Journal Requirements:

When submitting your revision, we need you to address these additional requirements.

1. Please ensure that your manuscript meets PLOS ONE's style requirements, including those for file naming. The PLOS ONE style templates can be found at
<https://journals.plos.org/plosone/s/file?id=wjVg/PLOSOne_formatting_sample_main_body.pdf> and
<https://journals.plos.org/plosone/s/file?id=ba62/PLOSOne_formatting_sample_title_authors_affiliations.pdf>

The style requirements are fulfilled.

2. In the online submission form, you indicated that [The data underlying this article is not publicly available due to potentially identifying and sensitive information (e.g., specific dates, cancer diagnoses and treatments, etc.). It will be shared on reasonable request to Lund University (contact via [ikvl-prefektadmin@med.lu.se](mailto:ikvl-prefektadmin@med.lu.se)).].
All PLOS journals now require all data underlying the findings described in their manuscript to be freely available to other researchers, either 1. In a public repository, 2. Within the manuscript itself, or 3. Uploaded as supplementary information.
This policy applies to all data except where public deposition would breach compliance with the protocol approved by your research ethics board. If your data cannot be made publicly available for ethical or legal reasons (e.g., public availability would compromise patient privacy), please explain your reasons on resubmission and your exemption request will be escalated for approval.

The Data Availability Statement has been updated in the submission system under the section Data Availability with the statement also added below. In addition, for the ethical application we assured that all data in manuscripts will be published for groups and not for individuals, being the reason for not making data freely available in a public repository. With our explanation, referring to legal reasons as well as sensitive information included in our data which could compromise patient privacy, we are kindly asking you to consider an approval for this exception.

Data Availability Statement:
The data underlying this article is not publicly available due to the General Data Protection Regulation (GDPR, a common European Union regulation), which regulates the handling of personal data and privacy protection in Sweden. Even though the data is pseudonymised, it can potentially be identifying with additional sensitive information (e.g., specific dates, cancer diagnoses and treatments, etc.), and therefore considered personal data under the GDPR. However, some data might be shared on reasonable request to Lund University (contact via [ikvl-prefektadmin@med.lu.se](mailto:ikvl-prefektadmin@med.lu.se)) if the receiver can guarantee a high level of data protection.

3. We note that you have included the phrase “data not shown” in your manuscript. Unfortunately, this does not meet our data sharing requirements. PLOS does not permit references to inaccessible data. We require that authors provide all relevant data within the paper, Supporting Information files, or in an acceptable, public repository. Please add a citation to support this phrase or upload the data that corresponds with these findings to a stable repository (such as Figshare or Dryad) and provide URLs, DOIs, or accession numbers that may be used to access these data. Or, if the data are not a core part of the research being presented in your study, we ask that you remove the phrase that refers to these data.

Since the information on fertility outcome for those below 40 years of age is not the core part of the data presented, we have removed the phrases under the paragraph “Fertility”:
- “We noted no difference regarding number of pregnancies and children born across the infertility risk groups for females <40 years of age (p=0.058 and p=0.083, respectively [data not presented]).”
- “as well as for those below 40 years (data not shown).”

4. We note that you have referenced (unpublished) on page 8, which has currently not yet been accepted for publication. Please remove this from your References and amend this to state in the body of your manuscript: (ie “Bewick et al. [Unpublished]”) as detailed online in our guide for authors
<http://journals.plos.org/plosone/s/submission-guidelines#loc-reference-style>

We have removed the word unpublished, and the reference is mentioned as “Vävnadsrådet [Unpublished]”.

5. Please review your reference list to ensure that it is complete and correct. If you have cited papers that have been retracted, please include the rationale for doing so in the manuscript text, or remove these references and replace them with relevant current references. Any changes to the reference list should be mentioned in the rebuttal letter that accompanies your revised manuscript. If you need to cite a retracted article, indicate the article’s retracted status in the References list and also include a citation and full reference for the retraction notice.

The reference list is complete and correct.

[Note: HTML markup is below. Please do not edit.]

Reviewers' comments:

Reviewers' Responses to Questions

**Comments to the Author**

1. Is the manuscript technically sound, and do the data support the conclusions?

The manuscript must describe a technically sound piece of scientific research with data that supports the conclusions. Experiments must have been conducted rigorously, with appropriate controls, replication, and sample sizes. The conclusions must be drawn appropriately based on the data presented.

Reviewer #1: Yes

Reviewer #2: Partly

Reviewer #3: Yes

2. Has the statistical analysis been performed appropriately and rigorously?

Reviewer #1: Yes

Reviewer #2: I Don't Know

Reviewer #3: Yes

3. Have the authors made all data underlying the findings in their manuscript fully available?

The [PLOS Data policy](http://www.plosone.org/static/policies.action#sharing) requires authors to make all data underlying the findings described in their manuscript fully available without restriction, with rare exception (please refer to the Data Availability Statement in the manuscript PDF file). The data should be provided as part of the manuscript or its supporting information, or deposited to a public repository. For example, in addition to summary statistics, the data points behind means, medians and variance measures should be available. If there are restrictions on publicly sharing data—e.g. participant privacy or use of data from a third party—those must be specified.

Reviewer #1: Yes

Reviewer #2: Yes

Reviewer #3: No

4. Is the manuscript presented in an intelligible fashion and written in standard English?

PLOS ONE does not copyedit accepted manuscripts, so the language in submitted articles must be clear, correct, and unambiguous. Any typographical or grammatical errors should be corrected at revision, so please note any specific errors here.

Reviewer #1: No

Reviewer #2: Yes

Reviewer #3: Yes

5. Review Comments to the Author

Please use the space provided to explain your answers to the questions above. You may also include additional comments for the author, including concerns about dual publication, research ethics, or publication ethics. (Please upload your review as an attachment if it exceeds 20,000 characters)

Reviewer #1: I read with great interest the Manuscript, which falls within the aim of this Journal.
In my honest opinion, the topic is interesting enough to attract the readers’ attention. Methodology is accurate and conclusions are supported by the data analysis. Nevertheless, authors should clarify some point and improve the discussion citing relevant and novel key articles about the topic.
Authors should consider the following recommendations:
- Manuscript should be further revised by a native English speaker.

The manuscript has been reviewed prior to the first submission by a native English speaker. This person has a PhD degree in the medical field and was hired through a professional certified translation agency. We contacted the agency once again and they have no further suggestions regarding the language. However, they suggested changing the word pubertal to pubescent, which has now been revised. The other two reviewers had no remarks considering the language. If there is any typographical and/or grammatical errors, please let us know and we will revise the manuscript accordingly.

- Inclusion/exclusion criteria should be better clarified.

The inclusion and exclusion criteria are described in the paragraph “Materials and methods – Subjects”. Here we have further clarified what type of rare cancer diagnoses were excluded, i.e., skin and thyroid cancer. Moreover, in the paragraph “Results – Participants” we have defined why 244 CCSs who met the inclusion criteria were excluded and added the following information: “i.e., diagnoses generally not represented in the childhood cancer population.”. Further exclusion criteria are also described in this paragraph: “202 females accepted participation, of whom 28 dropped out due to lack of time, three with ongoing pregnancy and four with pronounced disabilities were excluded, resulting in a total of 167 CCSs in this study.”.

We hope the inclusion and exclusion criteria are better clarified now.

- The authors have not adequately highlighted the strengths and limitations of their study. I suggest clarifying these points.

We have rewritten a sentence in the Discussion paragraph regarding limitations: “Lastly, we were unable to identify the gonadotoxic doses of radiotherapy and each chemotherapeutic agent due to heterogeneous treatments, multiple diagnoses, and limited sample size.”. In addition, we have also mentioned that the radiotherapy dose was in most cases reported as an interval rather than the exact dose.

A new section elucidating this study’s strengths has been added in the Discussion: “This study’s strengths were careful examination of the women for evaluation of ovarian function with ultrasound and serum markers and detailed treatment data for chemotherapeutic agents and radiotherapy doses for CCSs. Data was compared with a matched control group. We also had information about fertility for those who declined participation and could verify that the final study group of CCSs was not different regarding offspring.”

- What are the actual clinical implications of this study? It is important to report the results obtained by the authors in the context of clinical practice and to adequately highlight what contribution this study adds to the literature already existing on the topic and to future study perspectives.

The main clinical implication of this study is to emphasize the importance of fertility preservation prior to cancer treatment for those girls receiving highly gonadotoxic treatments. If the patient´s status or treatment start do not allow for fertility preservation, there is a need of follow-up at a young reproductive age in adulthood due to the potentially shortened reproductive window. This has been mentioned in the Discussion.

To better clarify the contribution of our study to the existing literature we added this in the Discussion: “There has been a discussion regarding CED cut-off, 6 or 8 g/m^2^, that should be recommended for fertility preservation. We believe our study supports the limit of 6 g/m^2^.”, and “To our knowledge, this is the first study evaluating the Swedish infertility risk classification and the PanCareLIFE recommendations in a patient cohort treated before fertility preservation was established among childhood cancer patients.”.

Regarding future study perspectives, the following text was included in the Discussion: “There is also a need for studies to identify genetic markers indicative of susceptibility to chemotherapy induced gonadal toxicity in order to give individualised fertility preservation recommendations.”

- The discussion should be further expanded, referring to the following PMID: 37798243, briefly discussing how the use of inositols can increase the chances of obtaining quantitatively and qualitatively good oocytes.

Inositols are now mentioned in the Discussion and the above reference is added: “New therapeutic options, like use of Inositols (i.e., insulin-sensitizing compounds) for assisted reproduction, might improve the chance of obtaining qualitatively good oocytes from CCSs at risk of ovarian dysfunction.”

Reviewer #2: I have read with great interest the proposed manuscript. It is well written and it addresses a very important topic. With this being said I have failed to see what is new and what this brings to the field in 2024. I think everyone agrees with the conclusions.

See above. We added a completion in the Discussion: “To our knowledge, this is the first study evaluating the Swedish infertility risk classification and the PanCareLIFE recommendations in a patient cohort treated before fertility preservation was established among childhood cancer patients.”

Reviewer #3: This study encompasses an interesting study cohort with sound data, especially considering the usual caveats for these long term follow-up cohorts. Its findings strengthen the current knowledge regarding different risk groups, and shows that the ovarian function/reserve is lower in the groups with greater risk. It is an interesting descriptions of their cohort. I’d like to challenge the group to also investigate what the accuracy is of the evaluated risk groups: perhaps using a ROC or sensitivity/specificity using one or more endpoints. Perhaps they can identify a cluster of women who showed a different outcome than expected based on their treatment risk group?

Thank you for your suggestion on using ROC. We have actually considered ROC, with endpoints such as POI, AFC <10 and unmeasurable AMH (i.e., <0.023 ng/ml), and CED as the predictor. To conduct this analysis, we had to exclude those who received abdominal radiotherapy, leaving only n=46 for the analysis. According to our statistician the number was too low for the ROC analysis. We were also unable to perform the analysis with radiotherapy doses as they were in most cases reported as an interval. However, we should mention that we have used ROC for this cohort in a previously published paper:

Nyström A, Mörse H, Nordlöf H, Wiebe K, Artman M, Øra I, et al. Anti-müllerian hormone compared with other ovarian markers after childhood cancer treatment. Acta Oncol. 2019;58(2):218-24. doi: 10.1080/0284186x.2018.1529423.

Regarding identification of unexpected outcomes for CCSs, we noticed one CCS with POI in the low-risk group. Another surprising result was the number of females given birth in the high-risk group (i.e., n=21 (57%)), which was comparable to the control women (54%). In the Discussion we have mentioned that the high-risk group showed no sign of affected fertility.

Minor details:
Methods: were there no women with a natural but irregular cycle? Were they excluded?

We did not exclude anyone based on information about menstrual cycles. We have added in the paragraph “Hormonal assay” that AMH for women with irregular cycles was analysed at a random day: “Fasting blood samples for AMH measurement were obtained during the early follicular phase (i.e., day 2–5 of the menstrual cycle) for females with regular cycles, and at a random day for those with irregular cycles, amenorrhea, or ongoing treatment with hormone replacement therapy (HRT) or oral contraceptives (OC).”

Were there more outliers perhaps in any of the groups regarding AMH and/or AFC?, possibly indicating PCOS?

Please see the boxplots below regarding outliers in the risk groups where women of all ages are represented. The group 7 is the control group. With an AMH level 10 ng/ml possibly suggestive of PCOS, we identified a total of 5 CCSs and 5 controls. Among these women 2/5 CCSs as well as 2/5 controls had given birth. We do not think that these figures possibly indicating PCOS, are of great importance for the infertility trends that we observed in the risk groups.

Results page 11 line 194: how do you know the number of offspring of the women who did not respond?

From the Swedish personal identification number, we could get data for the group of women who did not respond from the National Board of Social Affairs and Health, the Swedish Multi-Generation Registry. We added that in the text under the paragraph “Results – Participants”: “at the National Board of Social Affairs and Health”. The exact figures are also described in a previously published paper:

Nyström A, Mörse H, Nordlöf H, Wiebe K, Artman M, Øra I, et al. Anti-müllerian hormone compared with other ovarian markers after childhood cancer treatment. Acta Oncol. 2019;58(2):218-24. doi: 10.1080/0284186x.2018.1529423.

Page 12 line 219: what is the relevance of reporting that AMH levels did not differ in the groups with OC or HRT? The 20 patients on HRT must have relatively low AMH levels, if this does not lead to a difference in AMH levels between the groups one wonders a) why combine it with a completely different group on OC and b) if the power is anywhere near giving it an interesting significance.

The reason for combining OC and HRT in this analysis is that some studies report a reduction in AMH levels for women on OC. We checked the data for this as we could not adjust for the use of oestrogen when applying the Mann-Whitney U test or the Kruskal-Wallis test. We wanted to show that the number of CCSs and controls using OC or HRT were similar.

The results stated in this paragraph are not depicted in a table? That can be a choice, perhaps mentioning this.

This was only a minor analysis and does not really fit into any table that we presented in the manuscript. However, we chose to also include the AMH ranges for the groups: “1.8 (0.0–23.5) and 2.4 (0.0–14.3) ng/ml”.

6. PLOS authors have the option to publish the peer review history of their article ([what does this mean?](https://journals.plos.org/plosone/s/editorial-and-peer-review-process#loc-peer-review-history)). If published, this will include your full peer review and any attached files.


If you choose “no”, your identity will remain anonymous but your review may still be made public.


**Do you want your identity to be public for this peer review?** For information about this choice, including consent withdrawal, please see our [Privacy Policy](https://www.plos.org/privacy-policy).

Reviewer #1: No

Reviewer #2: No

Reviewer #3: No

[NOTE: If reviewer comments were submitted as an attachment file, they will be attached to this email and accessible via the submission site. Please log into your account, locate the manuscript record, and check for the action link "View Attachments". If this link does not appear, there are no attachment files.]

While revising your submission, please upload your figure files to the Preflight Analysis and Conversion Engine (PACE) digital diagnostic tool, <https://pacev2.apexcovantage.com/>. PACE helps ensure that figures meet PLOS requirements. To use PACE, you must first register as a user. Registration is free. Then, login and navigate to the UPLOAD tab, where you will find detailed instructions on how to use the tool. If you encounter any issues or have any questions when using PACE, please email PLOS at [figures@plos.org](mailto:figures@plos.org). Please note that Supporting Information files do not need this step.

Our figures have been uploaded in PACE and we have checked that they meet your requirements.
